# A single institution experience with palbociclib toxicity requiring dose modifications

**DOI:** 10.1007/s10549-017-4606-9

**Published:** 2017-12-07

**Authors:** Jun Gong, May Cho, Kim Wai Yu, James Waisman, Yuan Yuan, Joanne Mortimer

**Affiliations:** 1 Department of Medical Oncology and Therapeutics Research, City of Hope Comprehensive Cancer Center, 1500 E Duarte Rd, Duarte, CA 91010 USA; 2 Medical Oncology & Experimental Therapeutics, City of Hope Comprehensive Cancer Center, Building 51, Room 122, 1500 E Duarte Rd, Duarte, CA 91010 USA

**Keywords:** Palbociclib, Hormone receptor positive, Postmenopausal, Breast cancer, Neutropenia, Dose modification

## Abstract

**Purpose:**

Since the widespread implementation of adding palbociclib to endocrine therapy in clinical practice, myelosuppression is becoming increasingly recognized as a toxicity that may lead to dose modification. We aimed to characterize toxicities observed with palbociclib resulting in dose modifications and prescriber preferences in modifying palbociclib dosage in response to treatment-related toxicities outside the context of a clinical trial.

**Methods:**

We conducted a single institution, retrospective study of treatment-related adverse events (AEs) resulting in modifications in dose and schedule and the methods by which dose modifications occurred in patients with advanced hormone receptor (HR)-positive, human epidermal growth factor receptor 2 (HER2)-negative breast cancer receiving palbociclib and endocrine therapy.

**Results:**

From 2/2015 to 10/2016, 100 patients were identified for inclusion in this study. Treatment with palbociclib and endocrine therapy resulted in dose modifications in 38.0% of patients due to AEs with 18.4% requiring subsequent dose changes. Most palbociclib dose modifications occurred during the first 2 cycles. Grade 3–4 neutropenia accounted for 54.8% events of palbociclib dose modification. Most providers (65.8%) dose reduced palbociclib from 125 mg to 100 mg as their preferred method of dose modification, while others dose reduced from 125 mg to 75 mg (10.5%) and altered the schedule to 125 mg every other day (7.9%). A comparable rate of palbociclib dose modifications and subsequent dose changes were identified in an age ≥ 65 subgroup. In this group, dose adjustments were most commonly from grade 3–4 neutropenia, occurred mainly during cycle 1, and were most frequently addressed by dose reduction from 125 to 100 mg.

**Conclusions:**

Neutropenia remains the predominant cause for palbociclib dose modification and most modifications occur within the first two cycles. Older age (≥ 65) does not affect palbociclib tolerance. Our findings provide context outside of a clinical trial that inform ongoing studies evaluating the safety and feasibility of palbociclib-based therapies.

## Introduction

In February 2015, the CDK4/6 inhibitor, palbociclib, in combination with letrozole, was approved by the FDA as first-line treatment of metastatic postmenopausal, hormone receptor (HR)-positive breast cancer. Approval was based on the PALOMA 1 trial, which randomized 165 women to receive letrozole alone or letrozole in combination with palbociclib and demonstrated a significant improvement in overall response rate (ORR) and progression-free survival (PFS) in the combination study arm of 84 patients (letrozole plus palbociclib) [1]. The PALOMA 3 trial reported an improvement in PFS in both pre- and postmenopausal women when palbociclib was added to fulvestrant (plus goserelin in premenopausal women) in those who had received prior endocrine therapy [2]. Most recently, the PALOMA 2 trial confirmed the favorable results of PALOMA 1 in a larger population of patients [3]. These three studies established CDK4/6 inhibitors as an important class of drugs in the treatment of metastatic HR-positive/HER2-negative breast cancer.

The most common side effects of palbociclib are myelosuppression and fatigue. The PALOMA 2 study identified grade 3 and 4 neutropenia in 66.4% of patients receiving palbociclib and letrozole compared to 1.4% with letrozole alone [3]. The most common non-hematologic toxicities (all-grade) in the palbociclib + letrozole arm were fatigue (37.4%), nausea (35.1%), and arthralgia (33.3%). In PALOMA 1, the most common grade 1–2 adverse events (AEs) were fatigue (36%), anemia (29%), leucopenia (24%), and alopecia (22%) [1]. Grade 3–4 neutropenia was observed in 54% and leucopenia in 19% of patients. Although some degree of neutropenia was observed in 74% of patients receiving palbociclib compared with 4% treated with letrozole alone, no instances of febrile neutropenia were observed. In PALOMA 3, grade 3–4 AEs were more common in patients receiving palbociclib; neutropenia (65% in palbociclib + fulvestrant arm vs. 1% in placebo + fulvestrant arm), leucopenia (28% vs. 2%), anemia (3% vs. 2%), and thrombocytopenia (3% vs. 0%) [2]. All-grade neutropenia was observed in 81% of patients in the palbociclib group and 3% in the control group. Febrile neutropenia was rare and seen in only three patients receiving palbociclib + fulvestrant and one patient receiving placebo + fulvestrant.

According to the package labeling, it is recommended that prescribers monitor complete blood count prior to starting IBRANCE (palbociclib) at the beginning of each cycle, on day 15 of the first two cycles, and as clinically indicated. Dose interruption, dose reduction, or delay in starting treatment cycles is recommended for patients who develop grade 3 or 4 neutropenia [4]. As such, dose modifications may be made at a much lower threshold with palbociclib than what is conventionally used with chemotherapy. Given these unique recommendations for dose modification in this important new class of agents, we wanted to assess how these recommendations were being incorporated in practice outside the context of a clinical trial. The purpose of this study was to review non-clinical trial practice patterns for the use of palbociclib in patients with HR-positive, HER2-negative breast cancer.

## Methods

### Study population

This was a retrospective chart review study of patients primarily with locally advanced or metastatic (stage IV) HR-positive, HER2-negative breast cancer treated at the Breast Medical Oncology Clinic at City of Hope Comprehensive Cancer Center between February 2015 and October 2016. We included patients who were treated with the addition of palbociclib to endocrine therapy as described in PALOMA 1 and 3, irrespective of staging [1, 2]. On that note, 1 patient with stage II disease treated with a neoadjuvant strategy was allowed and included given that she was offered letrozole and palbociclib and was still in a non-clinical trial context. Patients were identified by the clinical pharmacist (KWY) who facilitated acquisition of palbociclib during this prespecified period. The study was approved by the Institutional Review Board (IRB) at City of Hope.

### Study design

All patients were initially prescribed palbociclib 125 mg oral daily for 21 consecutive days of a 28-day cycle based on registration trials [1, 2]. Peripheral blood was collected for a complete blood count (CBC) and absolute neutrophil count (ANC) upon initiation of palbociclib every 2 weeks on 2–3 occasions and thereafter based on provider discretion. Patient demographics including age, gender, menopausal status, tumor stage, number of previous treatments, and Eastern Cooperative Oncology Group (ECOG) performance status were recorded. Neutropenia was assessed by ANC every 2 weeks and dose modifications were recorded. Reasons other than neutropenia for dose medication, method of dose adjustment, and time to initial dose modification were also recorded. When possible, toxicities were classified based on Common Terminology Criteria for Adverse Events (CTCAE) version 4.03.

### Statistical analyses

All statistical analyses performed were descriptive. The sample size was determined by the total number of HR-positive, HER2-negative breast cancer patients treated at our institution with the addition of palbociclib to endocrine therapy within the prespecified study period. Categorical variables (e.g., sex) and ordinal variables (e.g., ECOG performance status) were organized and displayed in tabular form. All numeric values were expressed as whole numbers and percentages. When appropriate, a median and range was calculated for the age category. All descriptive statistics were conducted in Excel with associated formulas and functions. Descriptive statistics were performed for both the overall cohort (irrespective of age) and the cohort of patients aged ≥ 65 years.

## Results

### Study population

One hundred patients met criteria for inclusion in the study. Patient characteristics are summarized in Table 1. The median age was 61 (range 24–91 years) with 31 patients (31.0%) aged ≥ 65 years (range 65–91 years). All patients were postmenopausal or rendered postmenopausal with leuprolide. Most patients had an ECOG performance status of 0–1 (94.0%), had stage IV cancer (96.0%), and received ≤ 3 prior therapies (81.0%). Most prior therapies were chemotherapy (62.5%) while endocrine therapy comprised 27.3%. Of the 64 patients previously treated with chemotherapy, the majority (85.9%) had received 1–2 prior chemotherapy regimens, 9.4% received 3, and 4.7% received 4 or 5. Palbociclib was initiated most commonly in the setting of combination therapy with letrozole (69.0%) or fulvestrant (17.0%) (Table 1). A small percentage of prescribers included palbociclib in regimens containing both letrozole and fulvestrant (1.0%), letrozole, fulvestrant, and leuprolide (1.0%), letrozole and leuprolide (9.0%), and tamoxifen and leuprolide (2.0%).

**Table 1:** Patient characteristics

Characteristic (*n* = 100)	Frequency (%)
Age	Median (range)
	61 years (24–91 years)
Sex	
Female	99 (99.0%)
Male	1 (1.0%)
Menopausal status	
Premenopausal^a^	13 (13.0%)
Postmenopausal	86 (86.0%)
Stage^b^	
II^c^	1 (1.0%)
III^d^	3 (3.0%)
IV	96 (96.0%)
Number of previous systemic treatments^e^	
0–1	29 (29.0%)
2–3	52 (52.0%)
≥ 4	19 (19.0%)
Prior lines of therapy (*n* = 88)^f^	
Endocrine	24 (27.3%)
Chemotherapy	55 (62.5%)
Both	9 (10.2%)
Number of previous chemotherapy regimens (*n* = 64)^g^	
1	38 (59.4%)
2	17 (26.5%)
3	6 (9.4%)
4–5	3 (4.7%)
ECOG performance status	
0	62 (62.0%)
1	32 (32.0%)
2	6 (6.0%)
Endocrine therapy backbone (*n* = 100)	
Letrozole	69 (69.0%)
Fulvestrant	17 (17.0%)
Letrozole + fulvestrant	1 (1.0%)
Fulvestrant + leuprolide	1 (1.0%)
Letrozole + fulvestrant + leuprolide	1 (1.0%)
Letrozole + leuprolide	9 (9.0%)
Tamoxifen + leuprolide	2 (2.0%)

*ECOG* Eastern Cooperative Oncology Group

^a^All subsequently rendered postmenopausal with leuprolide

^b^According to the American Joint Committee on Cancer (AJCC) staging system

^c^As part of neoadjuvant endocrine therapy (not on clinical trial)

^d^As part of neoadjuvant endocrine therapy or for unresectable disease (not on clinical trial)

^e^Includes neoadjuvant/adjuvant chemotherapy and endocrine therapy

^f^Remaining patients presented with de novo metastatic disease

^g^Includes neoadjuvant and adjuvant chemotherapy

### Treatment-related adverse events leading to dose modifications

Of 100 patients receiving combination palbociclib and endocrine therapy, 38 patients (38.0%) required dose modifications in palbociclib (Table 2). Most dose modifications occurred during cycles 1–2 of therapy (81.6%). A smaller proportion of patients (10.6%) required dose modifications during cycles 3–4, and three patients (7.8%) needed changes in palbociclib dosing beyond cycle 5. Notably, seven patients (18.4%) receiving palbociclib required further dose adjustments beyond initial modifications in dosing.

**Table 2:** Reasons for dose modifications

Characteristic	Frequency (%)
Dose modification/delay needed (*n* = 100)	
No	62 (62.0%)
Yes	38 (38.0%)
Cycle during initial dose modification occurred (*n* = 38)	
1	27 (71.1%)
2	4 (10.5%)
3	2 (5.3%)
4	2 (5.3%)
≥ 5	3 (7.8%)
Subsequent dose reductions needed (*n* = 38)	
No	31 (81.6%)
Yes	7 (18.4%)
Reason for dose modification/delay (*n* = 42)^a^	
Neutropenia (all grades)	25 (59.6%)
Grade 1 neutropenia	1 (2.4%)
Grade 2 neutropenia	1 (2.4%)
Grade 3 neutropenia	14 (33.3%)
Grade 4 neutropenia	8 (19.1%)
Grade 4 neutropenia + grade 2 thrombocytopenia	1 (2.4%)
Anemia (grade 3)	1 (2.4%)
Thrombocytopenia (all grades)	5 (11.8%)
Grade 1 thrombocytopenia	2 (4.7%)
Grade 2 thrombocytopenia	2 (4.7%)
Grade 3 thrombocytopenia	1 (2.4%)
Fatigue (all grades)	2 (4.8%)
Grade 2 fatigue	1 (2.4%)
Grade 3 fatigue	1 (2.4%)
Mucositis (all grades)	4 (9.5%)
Grade 2 mucositis	3 (7.1%)
Grade 3 mucositis	1 (2.4%)
Pneumonitis (grade 3)	1 (2.4%)
Acute kidney injury (grade 1)	1 (2.4%)
Septic shock	1 (2.4%)
Anticipated tolerance (age)	2 (4.7%)
Method of dose modification (*n* = 38)	
Dose reduction to 100 mg	25 (65.8%)
Dose reduction to 75 mg	4 (10.5%)
Dose delay/hold indefinitely or resume at 125 mg^b^	6 (15.8%)
Schedule change	
Every other day 125 mg	3 (7.9%)

^a^Each adverse event resulting in dose modification included (multiple dose modifications can occur in any one patient)

^b^Two of six patients had palbociclib held indefinitely

The reasons for modifications in palbociclib dose and schedule are shown in Table 2. Out of 42 events leading to dose modifications in palbociclib, the most frequent were treatment-related AEs of grade 3–4 neutropenia (54.8%, 1 with concurrent grade 2 thrombocytopenia), grade 1–3 thrombocytopenia (11.8%), grade 2–3 mucositis (9.5%), and grade 2–3 fatigue (4.8%). Two patients had their starting dose of palbociclib reduced for anticipated tolerance due to age.

An event of septic shock occurred in a 62-year-old postmenopausal female with metastatic HR-positive, HER2-negative, breast cancer on first-line palbociclib (125 mg) and letrozole. During her first cycle, her course was complicated by possibly treatment-related pancytopenia and sepsis. Although her palbociclib was held prior to her sepsis, she ultimately died (received 1 cycle of palbociclib overall). Her death was reported as possibly treatment-related to palbociclib.

A unique treatment-related AE of pneumonitis was observed in a 72-year-old postmenopausal female with HR-positive metastatic breast cancer on second-line palbociclib (125 mg) and fulvestrant. By cycle 8, she was hospitalized for worsening dyspnea; and a CT scan identified a similar extent of metastatic disease but new bilateral pulmonary ground-glass opacities suggestive of an inflammatory process. Extensive infectious work-up returned negative and she was stabilized on supportive care and supplemental oxygen. She was subsequently discharged on home oxygen and her palbociclib was discontinued for suspected treatment-related grade 3 pneumonitis. Her pneumonitis completely resolved within 3 months of palbociclib discontinuation.

Of interest, three patients on first-line palbociclib (125 mg) and letrozole and 1 patient on second-line palbociclib (125 mg) and fulvestrant experienced grade 2 and 3 treatment-related mucositis, respectively, leading to dose reductions in palbociclib. The manifestations of mucositis included mouth pain without overt ulceration, pain with few (usually less than 3) punctate (1–2 mm) irregular-shaped ulcers within the buccal mucosa, or pain with multiple 1–2 mm ulcerations on the lower lip and swelling of the lips and tongue that developed as early as after cycle 1 to as late as cycle 5. Two of the four cases required 2 dose reductions to 75 mg (in 1 instance, 1 of the 2 dose reductions was unrelated to mucositis) and the other 2 required 1 dose reduction to 100 mg. Mucositis generally improved with dose reductions and supportive care though only one case (without overt ulceration) was able to have palbociclib escalated back to 125 mg per provider discretion.

### Methods of dose modification

The methods by which prescribers modified palbociclib dosing in response to toxicities are shown in Table 2. Dose reduction from the full dose of 125 mg daily palbociclib to 100 mg daily was the most frequent dose modification (65.8%) among the 38 patients who required dose modifications. Less common dose modification strategies included holding palbociclib indefinitely or resuming at full dose 125 mg daily (usually by the start of the next cycle) when toxicity improved (15.8%), reducing palbociclib from 125 mg to 75 mg daily (10.5%), and altering the schedule of palbociclib administration to 125 mg every other day (7.9%) in response to treatment-related AEs.

### Age ≥ 65 subgroup dose modifications

In the cohort of 31 patients ≥ 65 years of age, patient characteristics and reasons for palbociclib dose modifications in those requiring modifications are shown in Table 3. Most patients (87.1%) were treated with ≤ 3 prior therapies. Of the prior lines of therapy, 57.2% were chemotherapy and 32.1% were endocrine therapy. Of the 19 having received previous chemotherapy, 57.9% received 1, 36.8% received 2, and 5.3% received 3 prior chemotherapy regimens. Similar to the overall cohort, most patients had an ECOG performance status of 0–1 (96.8%). Palbociclib was most frequently added to a backbone of letrozole (80.6%), while palbociclib was added to fulvestrant in 19.4% patients. Dose modifications were required in 38.7% of patients in this subgroup with 91.7% of these dose modifications occurring during the first cycle of palbociclib. Of the 15 events leading to dose modifications in palbociclib, the most frequent were treatment-related AEs of grade 3–4 neutropenia (46.6%), followed by treatment-related grade 2–3 mucositis (13.3%), and anticipated tolerance due to age (13.3%). The most common method of dose modification was reducing the full dose of 125 mg daily palbociclib to 100 mg daily (50.0%) followed by reduction from 125 to 75 mg (33.3%), and dose delay/hold indefinitely or resume at 125 mg (16.7%). Three patients in this subgroup required subsequent dose modifications (25.0%).

**Table 3:** Age ≥ 65 subgroup and dose modifications

Characteristic (*n* = 31)	Frequency (%)
Range (years)	65–91
Sex	
Female	30 (96.8%)
Male	1 (3.2%)
Stage^a^	
III^b^	2 (6.5%)
IV	29 (93.5%)
Number of previous systemic treatments^c^	
0–1	9 (29.0%)
2–3	18 (58.1%)
≥ 4	4 (12.9%)
Prior lines of therapy (*n* = 28)	
Endocrine	9 (32.1%)
Chemotherapy	16 (57.2%)
Both	3 (10.7%)
Number of previous chemotherapy regimens (*n* = 19)^d^	
1	11 (57.9%)
2	7 (36.8%)
3	1 (5.3%)
ECOG performance status	
0	18 (58.1%)
1	12 (38.7%)
2	1 (3.2%)
Endocrine therapy backbone (*n* = 31)	
Letrozole	25 (80.6%)
Fulvestrant	6 (19.4%)
Dose modification/delay needed (*n* = 31)	
No	19 (61.3%)
Yes	12 (38.7%)
Cycle during initial dose modification occurred (*n* = 12)	
1	11 (91.7%)
4	1 (8.3%)
Subsequent dose reductions needed (*n* = 12)	
No	9 (75.0%)
Yes	3 (25.0%)
Reason for dose modification/delay (*n* = 15)^e^	
Neutropenia (all grades)	8 (53.3%)
Grade 2 neutropenia	1 (6.7%)
Grade 3 neutropenia	5 (33.3%)
Grade 4 neutropenia	2 (13.3%)
Mucositis (all grades)	2 (13.4%)
Grade 2 mucositis	1 (6.7%)
Grade 3 mucositis	1 (6.7%)
Thrombocytopenia (grade 2)	1 (6.7%)
Pneumonitis (grade 3)	1 (6.7%)
Acute kidney injury (grade 1)	1 (6.7%)
Anticipated tolerance (age)	2 (13.3%)
Method of dose modification (*n* = 12)	
Dose reduction to 100 mg	6 (50.0%)
Dose reduction to 75 mg	4 (33.3%)
Dose delay/hold indefinitely or resume at 125 mg^f^	2 (16.7%)

*ECOG* Eastern Cooperative Oncology Group

^a^According to the American Joint Committee on Cancer (AJCC) staging system

^b^As part of neoadjuvant endocrine therapy or for unresectable disease (not on clinical trial)

^c^Includes neoadjuvant/adjuvant chemotherapy and endocrine therapy

^d^Includes neoadjuvant and adjuvant chemotherapy

^e^Each adverse event resulting in dose modification included (multiple dose modifications can occur in any one patient)

^f^One patient had palbociclib held indefinitely

## Discussion

Palbociclib was initially approved on the basis of a small number of patients treated on the PALOMA 1 study. The PALOMA 2 and PALOMA 3 trials included over 700 patients and confirmed grade 3 and 4 neutropenia as the most common side effect from palbociclib and endocrine therapy [2, 3]. Patients who participate in registration trails are generally stringently selected for performance status, age, and limited prior therapies based on prespecified inclusion criteria [5]. A non-clinical trial experience with new agents is important as drug toxicities in routine clinical practice may be more common or more severe than those observed in clinical trial settings. The incidence of grade 3 and 4 neutropenia in our population (54.8%) was comparable to the incidence reported in the aforementioned studies. Although we recognize that our study was conducted through a specialized breast medical oncology clinic and may not be entirely representative of community oncology practices, we also observed the use of non-standard dose modification schemes for palbociclib that are reflective of the variability in real-world individual provider practice patterns. For example, the package insert recommends providers to hold palbociclib for grade 3 neutropenia on day 1 of each cycle, check a CBC in 1 week, and resume at same dose for next cycle if recovered to grade ≤ 2; dose reductions to next lower dose are recommended for grade 3 neutropenia > 1 week, recurrent grade 3 neutropenia, grade 3 neutropenia with fever and/or infection, or grade 4 neutropenia [4]. For grade 1–2 hematologic and non-hematologic toxicities, no dose modifications are required. For grade ≥ 3 non-hematologic toxicities, it is recommended to hold palbociclib until symptoms resolve to grade 1–2 and resume at next lower dose. In our study, we saw dose modifications for grade 1–2 neutropenia (4.8%), grade 1–2 thrombocytopenia (9.4%), grade 2 fatigue (2.4%), grade 2 mucositis (7.1%), grade 1 renal injury (2.4%), and for anticipated tolerance due to age (4.7%). Of the six patients (15.8%) managed by holding palbociclib and resuming by next cycle or holding indefinitely, two of these patients ultimately required dose adjustments. We also observed modifications by way of every other day dosing and direct dose reduction to 75 mg daily (Table 2) that altogether underscore variability in management practices per provider discretion.

Of note, another CDK4/6 inhibitor, ribociclib, was recently approved in the first-line treatment of postmenopausal, HR-positive/HER2-negative advanced breast cancer based on superior median PFS with ribociclib + letrozole (not reached) compared to placebo + letrozole (median PFS 14.7 months, hazard ratio 0.56 (95% CI 0.43–0.72, *p* < 0.0001) in the phase III MONALEESA-2 trial [6]. Also recently approved was the CDK4/6 inhibitor, abemaciclib, as monotherapy or in combination with fulvestrant in the treatment of HR-positive, HER2-negative advanced or metastatic breast cancer with disease progression following endocrine therapy and, if applicable, prior chemotherapy based on the phase II MONARCH 1 and phase III MONARCH 2 trials [7, 8]. MONARCH 1 was an open-label, single-arm trial investigating single-agent abemaciclib 200 mg oral twice daily until disease progression and showed an ORR of 19.7% (95% CI 13.3–27.5), which was its primary objective [7]. Single-agent abemaciclib was associated with a grade 3–4 neutropenia rate of 26.8%. The randomized, placebo-controlled phase III MONARCH 2 investigated abemaciclib 150 mg oral twice daily with fulvestrant (standard dosing) versus fulvestrant alone and showed a superior median PFS of 16.4 months in the combination arm versus 9.3 months in the fulvestrant alone arm (HR 0.553, 95% CI 0.449–0.681, *p* < 0.0000001) [8]. All-grade treatment-emergent neutropenia was more common in the combination arm vs. fulvestrant alone (46.0% vs. 4.0%) though diarrhea was the most frequent AE (86.4% vs. 24.7%). The rate of grade 3–4 neutropenia in the ribociclib arm was 59.3%, which is comparable to the 54–66.4% grade 3–4 neutropenia rate observed in the palbociclib arms of the PALOMA studies [1–3]. Further studies are warranted to validate if our data are generalizable to other CDK4/6 inhibitors in routine clinical practice.

On analysis of our age ≥ 65 subgroup (*n* = 31), we found comparable rates of initial dose modifications (38.7%) and subsequent dose changes (25.0%) to the overall cohort. Similarly, most dose modifications occurred early in the treatment course (91.7% during cycle 1) and were most frequently a result of grade 3–4 neutropenia (46.6%), grade 2–3 mucositis (13.3%), or anticipated tolerance due to age (13.3%). Of the 12 patients that required dose modifications in palbociclib, the most common method was reduction to 100 mg (50.0%) followed by reduction to 75 mg (33.3%). This is consistent with findings from a recent pooled analysis of PALOMA trials that included 221 patients aged ≥ 65 to 74 and 83 patients aged ≥ 75 out of 872 patients treated with palbociclib + letrozole or fulvestrant [9]. They showed that the incidence of AEs and palbociclib discontinuations were similar in the overall cohort and in patients aged ≥ 65 to 74 and ≥ 75. The incidence of all-grade and grade 3–4 neutropenia was also similar across all age groups. Furthermore, improvements in efficacy endpoints were seen across all age groups without any clinically relevant differences in pharmacokinetics. Our results support their conclusions that there was no difference in tolerance of palbociclib + endocrine therapy between patients aged ≥ 65 and other patients with HR-positive, HER2-negative advanced breast cancer; and a dose adjustment based on age is not required.

It is anticipated that new side effects will be identified in the postmarketing period of palbociclib. It is estimated that half of all serious AEs are only completely realized more than seven years after FDA approval [10]. The incidence of stomatitis was 11–15% in the PALOMA studies with < 1% grade 3–4 [1, 3, 11]. The manifestations of mucositis/stomatitis in our patients varied, but typically presented as tiny mucosal lesions that did not interfere with oral intake and spontaneously resolved. Absence of overt ulcerations, but rather complaints of an inflamed, sore mouth were also seen. More severe cases involved swelling of the tongue and lips and impairment in the ability to consume food. The occurrence varied in onset as early as cycle 1 to a relatively protracted course beyond cycle 5 requiring subsequent dose reductions and occurred often in the setting of grade ≥ 2 neutropenia. We also described a unique case of palbociclib-related pneumonitis observed in a 72-year-old patient who was started on second-line palbociclib (125 mg) and fulvestrant. Along with supportive care, her pneumonitis resolved within 3 months of discontinuation of palbociclib. To our knowledge, this is the first case described in the literature of palbociclib-associated pneumonitis. The identification of grade 2–3 mucositis comprising 9.5% of AEs leading to dose modifications in our cohort also highlights a poorly characterized but relevant AE associated with palbociclib. Mucositis is now recognized as a side effect of palbociclib and has been added to the package insert.

In conclusion, the addition of palbociclib to endocrine therapy in advanced HR-positive, HER2-negative breast cancer results in dose modifications in 38.0% of patients due to AEs with 18.4% requiring subsequent dose changes. Most palbociclib dose modifications occurred during the first two cycles. The most common reason for palbociclib dose modifications was grade 3–4 neutropenia (54.8%). Most providers (65.8%) dose-reduced palbociclib from 125 mg to 100 mg as their preferred method of dose modification when needed. Grade 2–3 mucositis comprised 9.5% of AEs leading to dose modifications, and we described the first case of palbociclib-related pneumonitis; both toxicities are in need of further recognition and characterization. Our findings provide context outside of a clinical trial that can inform ongoing studies of larger size and prospective design evaluating the safety and feasibility of palbociclib-based therapies. This study is limited by its retrospective design. However, our data provides useful insights into the prescribing patterns of physicians while they become comfortable with use of a new agent.
